# A multimodal approach to cardiovascular risk stratification in patients with type 2 diabetes incorporating retinal, genomic and clinical features

**DOI:** 10.1038/s41598-019-40403-1

**Published:** 2019-03-05

**Authors:** Ahmed E. Fetit, Alexander S. Doney, Stephen Hogg, Ruixuan Wang, Tom MacGillivray, Joanna M. Wardlaw, Fergus N. Doubal, Gareth J. McKay, Stephen McKenna, Emanuele Trucco

**Affiliations:** 1 VAMPIRE project, Computer Vision and Image Processing Group, School of Science and Engineering (Computing), University of Dundee, Dundee, Scotland United Kingdom; 2 Ninewells Hospital and Medical School, University of Dundee, Dundee, Scotland United Kingdom; 3 VAMPIRE project, Centre for Clinical Brain Sciences, University of Edinburgh, Edinburgh, Scotland United Kingdom; 4 Centre for Clinical Brain Sciences, University of Edinburgh, Edinburgh, Scotland United Kingdom; 5 Centre for Public Health, Queen’s University Belfast, Belfast, Northern Ireland United Kingdom

## Abstract

Cardiovascular diseases are a public health concern; they remain the leading cause of morbidity and mortality in patients with type 2 diabetes. Phenotypic information available from retinal fundus images and clinical measurements, in addition to genomic data, can identify relevant biomarkers of cardiovascular health. In this study, we assessed whether such biomarkers stratified risks of major adverse cardiac events (MACE). A retrospective analysis was carried out on an extract from the Tayside GoDARTS bioresource of participants with type 2 diabetes (n = 3,891). A total of 519 features were incorporated, summarising morphometric properties of the retinal vasculature, various single nucleotide polymorphisms (SNPs), as well as routine clinical measurements. After imputing missing features, a predictive model was developed on a randomly sampled set (n = 2,918) using L1-regularised logistic regression (lasso). The model was evaluated on an independent set (n = 973) and its performance associated with overall hazard rate after censoring (log-rank *p* < 0.0001), suggesting that multimodal features were able to capture important knowledge for MACE risk assessment. We further showed through a bootstrap analysis that all three sources of information (retinal, genetic, routine clinical) offer robust signal. Particularly robust features included: tortuousity, width gradient, and branching point retinal groupings; SNPs known to be associated with blood pressure and cardiovascular phenotypic traits; age at imaging; clinical measurements such as blood pressure and high density lipoprotein. This novel approach could be used for fast and sensitive determination of future risks associated with MACE.

## Introduction

Cardiovascular diseases (CVD) remain the leading cause of morbidity and mortality in patients with type 2 diabetes and are largely preventable. A key step towards prevention is accurate stratification of risk, allowing appropriate targeting of maximally effective intervention strategies. Patients with type 2 diabetes undergo regular eye screening to manage risk of sight-threatening diabetic retinopathy. The retina may also represent a source of information indicative of global vascular health; a wide range of studies report associations between retinal features and cardiovascular risk factors. These include the Rotterdam Study^1^, the Cardiovascular Health Study^2^ and the meta-analysis by McGeechan *et al*.^3^ on over 22,000 participants from 6 population-based studies. This motivates the study of standard retinal photographs obtained through eye screening as a source of phenotypic biomarkers of risk of cardiovascular disease.

Recently, Poplin *et al*.^4^ analysed datasets from the UK Biobank^5^ and EyePACS^6^ cohorts using deep learning methods. They trained neural networks to predict known risk factors such as smoking status and systolic blood pressure from retinal images. Additionally, the study revealed that it is possible to predict Major Adverse Cardiovascular Events (MACE) from retinal images using deep learning models, achieving a 0.7 area under the receiver operating characteristic curve. Whilst the analysis was carried out on large cohorts (>48,000 patients, UK Biobank; >236,000 patients, EyePACS), the number of patients known to have experienced MACE events was relatively small (631 events, UK Biobank) and MACE information was not available for the EyePACS data. Furthermore, while deep neural networks can improve prediction through the use of non-linear feature hierarchies and very large cohorts where available, the clinical interpretability of such models remains uncertain.

With the currently increasing emphasis on interpretability of artificial intelligence systems^7^, studying the role of clinically interpretable retinal features such as vessel calibre and tortuosity is essential. In contrast to prediction via deep learning, our approach yields features with direct clinical interpretability while still achieving significant risk stratification. Phenotypic information available from retinal fundus images and routine clinical measurements, in addition to genomic data offer complementary perspectives on disease risks^8^; incorporating them in a multimodal approach may provide a more nuanced assessment of disease risk and stratified therapeutic approaches to reduce risk. We thus describe a computational approach combining measurements from retinal fundus images, genomic and clinical data to generate a multimodal classifier for MACE in patients with type 2 diabetes from Tayside, Scotland.

## Methods

### Analysis dataset

Data from 3,891 individuals with type 2 diabetes were selected from the GoDARTS bioresource^9^. Participants underwent regular diabetic retinopathy screening and had digital fundus images that matched our quality criteria for semi-automated analysis of retinal vascular features with several clinical outcomes^10^. VAMPIRE 3.1 software (Vascular Assessment and Measurement Platform for Images of the Retina, Universities of Dundee and Edinburgh, Scotland, UK)^11–13^ was used to semi-automatically measure features with a direct clinical interpretation. Features were measured from standard pre-defined annular zones, following well-established protocols^11^, and included optic disc (OD) radius, central retinal arteriolar equivalent (CRAE), central retinal venular equivalent (CRVE), retinal arterio-venule-ratio (AVR), tortuosity of arteries (tortA) and veins (tortV), by retinal zone, quadrant, vessel generation and vessel type (artery or vein). A total of 157 retinal features were available per image. Readers are referred to Supplementary Material for a detailed explanation of the different retinal feature sub-categories. Two trained operators (SH and RW) performed the measurements following a standard, validated protocol for VAMPIRE. Training for each operator was carried out over two sessions:^11^ an introductory session where the protocols and software were presented and familiarity with them gained through practice on a demonstration image set (n = 20, one day); and an assessment session where competency in operation was assessed on a testing image set (n = 20, one day). Training lasted for approximately two days in total and was followed by periodic re-validation sessions.

We used a validated data linkage algorithm on anonymised electronic medical records of GoDARTS participants. The median of each clinical measure for a 3-year period prior to the date of the fundus photograph was obtained. Clinical measures were diastolic and systolic blood pressures adjusted for blood pressure lowering drugs, total cholesterol, high density lipoprotein (HDL) cholesterol, triglycerides levels and glycated haemoglobin (Table 1). Additionally, we incorporated information on the median number of blood pressure lowering drugs, smoking history, cardiovascular disease history, duration of diabetes, age at imaging, and sex. A total of 343 single nucleotide polymorphisms (SNPs) were also included. These were selected from the GoDARTS genotype database and consisted of available SNPs that had been identified in previous genome-wide association studies for cardiovascular disease^14^, blood pressure^15^ and Alzheimer’s disease^16^. Weighted genetic risk scores for each phenotype were constructed using the relevant SNPs. These risk scores were all included in the analysis, in addition to the entire set of individual SNPs. MACE was defined as hospitalisation for myocardial infarction or stroke, or cardiovascular death. This was determined through linkage with hospital admission and cause of death records similar to previously reported studies^10^. Participants were censored at date of non-cardiovascular death or last available date of follow-up.

**Table 1:** Participant characteristics (mean values) in the development and clinical validation sets.

Characteristics	Model development set		Clinical validation set	
	MACE	No MACE	MACE	No MACE
Number of patients	910	2,008	309	664
Age at imaging (years)	72.45	68.13	72.38	68.32
Sex (% female)	43	48	45	48
Time to event or censoring (years)	3.37	7.38	3.69	7.42
OD radius (pixels)	198.7	195.5	199.1	196.2
CRAE (pixels)	32.4	32.3	32.6	32.3
CRVE (pixels)	42.7	42.7	43.0	42.7
Log of tortA	10.2 × 10^−5^	10.3 × 10^−5^	10.1 × 10^−5^	9.92 × 10^−5^
Log of tortV	6.9 × 10^−5^	6.4 × 10^−5^	7.14 × 10^−5^	6.4 × 10^-5^
AVR	0.76	0.76	0.76	0.76
CVD gene score	4.47	4.38	4.44	4.41
Corrected systolic blood pressure (mmHg)	141.05	141.30	141.79	142.47
Corrected diastolic blood pressure (mmHg)	76.54	78.95	77.29	79.43
Cholesterol levels (mmol/L)	4.25	4.36	4.25	4.36
High density lipoproteins (mmol/L)	1.30	1.36	1.32	1.38
Log Triglycerides (mmol/L)	2.19	2.10	2.14	2.08
History of CVD (% yes)	52	23	51	20
History of smoking (%yes)	81	72	79	73

Retinal length measurements are in pixels to avoid the uncertainty introduced by commonly used pixel-micron conversion factors^30^. Differences in image size and resolution are taken into account by VAMPIRE^11–13^. OD: optic disc; CRAE: central retinal arteriolar equivalent; CRVE: central retinal venular equivalent; tortA: tortuosity of arteries, tortV: tortuosity of veins, AVR: retinal arterio-venule-ratio; CVD: cardiovascular disease.

### Analysis pipeline

#### Sampling and imputation of data

The dataset was rather heterogeneous as only 239 participants had no missing features. A total of 2,893 participants had between 1 and 61 missing features each (median missing retinal features = 0; genomic = 2; clinical = 0). This was higher in the remaining 759 participants, who each had between 270 and 384 missing features (median missing retinal features = 0; genomic = 347; clinical = 0). Following concatenation of retinal, genomic and clinical data, 75% of the cohort was sampled at random and used to build and fine-tune the model (model development set). The remaining 25% was retained for model validation (clinical validation set). A k-nearest neighbour algorithm (k = 10) was used to impute missing features using the *knn.impute* function of the *bnstruct* package in R^17^. In essence, the algorithm obtains imputed values from similar participant profiles; all available features were used to search for the neighbours. For continuous features, the neighbours’ median value over the set of similar profiles was used, whilst for categorical features the mode was used. This step was blinded to the participant’s MACE outcome to avoid leakage of class information into the predictive model. Imputation was undertaken separately for the development and clinical validation sets.

#### Computation of the multimodal MACE classifier

The model development set was used to build classifiers for predicting the binary outcome of MACE onset before censoring. The well-established L_1_-regularised logistic regression (lasso)^18^ performed simultaneous feature selection and model estimation. Implementation used the R *glmnet* package^19^. No-MACE participants occurred 2.2 times as often as MACE participants in the dataset (see Table 1). To account for this class imbalance, weights were assigned to each observation (the *MACE: no-MACE* assigned observation weights were *2.2:1*). The λ parameter, which controls the strength of regularisation and hence model sparsity, was fine-tuned using 10-fold cross-validation. The value resulting in the lowest binomial deviance, λ_min_, was identified and used to train a model on the entire development set. A second value, λ_1SE,_ is also of interest as it corresponds to the most regularised model leading to a binomial deviance within one standard error of that obtained at λ_min_, usually involving fewer features (given the less strict requirement on the binomial deviance). We performed risk stratification using a λ_min_-based model’s prediction of an individual’s probability of MACE before censoring. We further explored whether a more compact λ_1SE_-based model could achieve similar performance outcomes.

#### Evaluation of performance on the clinical validation set

A tuned λ_min_-based model was validated on the clinical validation set (random 25% retained subset from the original cohort). The output probability predicted by the model was used to stratify patients into two groups, high-risk and low-risk, and to generate Kaplan-Meier plots. Stratification was undertaken using a predefined threshold identified by a 10-fold cross-validation stage, specifically the mean of the model output probabilities across participants. To assess statistical significance between both groups, a log-rank p value was computed using the *survival* package in R^20^. This process was repeated using a λ_1SE_ -based model^21^.

#### Evaluation of feature robustness using bootstrap

Different data samples give rise to different feature sets being selected when computing the classifier. We performed a bootstrap analysis to assess how likely features were to be selected over a large number of randomly selected training sets^22^. The frequency with which a feature or feature set was selected across the bootstraps was used as a proxy measure of feature robustness. A total of 500 bootstrap trials was carried out on the development set using λ_min_, and the proportion of times each feature had a non-zero weight was recorded, providing a measure of how likely each feature is to be selected. Binomial deviance was calculated across the 500 bootstraps, together with the corresponding 95% confidence intervals (CI). A total of 910 samples from each class was included in every trial to ensure class balancing.

In addition to individual feature occurrence, we computed the frequencies with which at least one retinal, genomic and clinical feature was selected across the bootstraps. Retinal features can be broadly divided into six sub-categories: tortuosity (108 features), width gradient (16), branching point (18), fractal analysis (6), OD-based (2), and Zone B width (5) features. Features within each sub-category may be highly correlated, and as such we were interested in the frequencies with which at least one feature from each retinal sub-category was selected across the bootstraps. Readers are referred to Supplementary Material for a detailed explanation of the different retinal feature sub-categories.

## Results

### Participant baseline characteristics

A total of 1,219 individuals were recorded as undergoing MACE during the follow-up period. The mean and median times to MACE following retinal imaging were 3.45 and 2.99 years respectively with a standard deviation of 2.48 years. The mean and median ages for these participants at imaging were 72.43 and 73.54 years respectively; 528 participants were female and 691 were male.

For the remaining participants (no-MACE), points of right censoring ranged from 0.05 years to 10.95 years post image-capture. The mean and median times to censoring were 7.39 and 8.81 years, respectively; standard deviation was 2.65 years. For no-MACE participants, the mean and median age at imaging were 68.17 and 69.20 years respectively; 1,293 participants were female and 1,379 were male. A breakdown of participant demographics for the development and clinical validation sets is shown in Table 1.

### Model development and tuning

**Figure 1:**
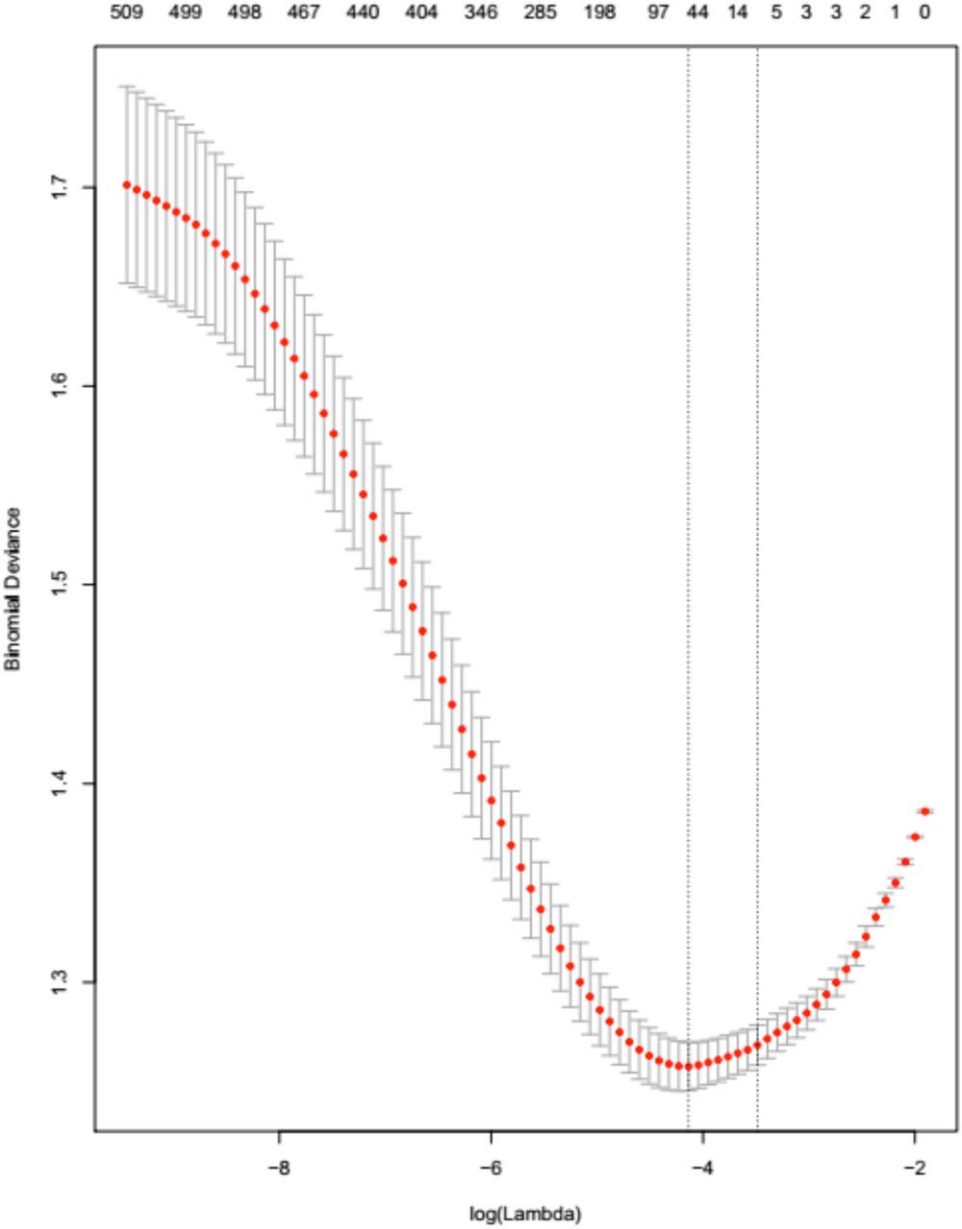
The results of repeated 10-fold cross-validation (CV) experiments on the development set, showing how variation in λ affects binomial deviance. The numbers at the top of the figure indicate numbers of features retained within the regularised models. Interval bars represent standard deviation. The vertical line to the left represents λ_min_, whereas the one to the right represents λ_1SE_.

The cross-validation curve for the development set is indicated by the red dotted line in Fig. 1. Upper and lower standard deviation curves (error bars) are also plotted. Increasing the extent of regularisation reduced binomial deviance until a model that retained 51 features was reached; beyond that point, regularisation resulted in increased binomial deviance. Two selected λ values are indicated by the vertical dotted lines: λ_min_ (minimum binomial deviance) and λ_1SE_ (binomial deviance within 1 SE of the minimum).

As can be seen in Table 2, the λ_min_-based model had 51 features from all three categories: retinal, genetic and clinical. Selected retinal features comprised optic disc radius, venular gradient width, venular fractal dimension, as well as various tortuosity measures. Of the genetic features included, 34 SNPs were selected. As per the *Ensembl* genome browser (Human GRCh38.p12 assembly), these variants had been previously shown to be associated with a variety of phenotypic traits that include cardiovascular disease, blood pressure, and Alzheimer’s disease. Examples of selected SNPs and their known phenotypic associations include rs34923683: pulse pressure measurement; rs9549328: systolic blood pressure; rs687621: cholesterol; rs12921187: diastolic blood pressure; rs12413409: coronary heart disease; rs2048327: coronary heart disease; and rs11218343: Alzheimer’s disease; (refer to Table 2 for a complete list of SNPs selected by the λ_min_-based model). Noteworthy, of the 3 composite scores analysed, only the cardiovascular gene score was selected. Finally, age at imaging and a variety of clinical measurements were selected by the model, namely: number of blood pressure lowering drugs taken, history of smoking, evidence of CVD before imaging, diastolic blood pressure, high density lipoprotein, glycated haemoglobin, triglycerides, and duration of diabetes.

**Table 2:** 51 features assigned non-zero coefficients; these constitute a regularised model using λ_min_ on the entire development set. For the λ_1SE_ -based model, only 7 features were retained.

Category	Non-zero coefficient features	
	Using λ_min_	Using λ_1se_
Retinal	• odradiuspx • tortq4g1vstd • tortq2g1vmed • tortq2g1a • tortq1g1vmed • gradq4vhermite • d1v	• None selected
SNPs	• rs34923683 • rs3752728 • rs9549328 • rs687621 • rs12921187 • rs11218343 • rs12413409 • rs2048327 • rs2014408 • rs1878406 • rs13359291 • rs2493292 • rs7136259 • rs79089478 • rs8258 • rs7248104 • rs419076 • rs2895811 • rs983392 • rs11203042 • rs1530440 • rs1563788 • rs2240736 • rs12941318 • rs6686889 • rs7126805 • rs12906962 • rs10850411 • rs10792832 • rs7515635 • rs2291435 • rs449789 • rs4308 • rs200999181	• None selected
Gene scores	• CVD gene score	• CVD gene score
Clinical	• Number of blood pressure lowering drugs taken • History of smoking • Evidence of CVD before imaging • Diastolic blood pressure • High density lipoprotein • Age at imaging • Glycated Haemoglobin • Triglycerides • Duration of diabetes	• Number of blood pressure lowering drugs taken • History of smoking • Evidence of CVD before imaging • Diastolic blood pressure • High density lipoprotein • Age at imaging

CVD: cardiovascular disease. Readers are referred to Supplementary Material for a detailed explanation of retinal features computed by VAMPIRE.

Notably, whilst the λ_min_-based model had 51 features, λ_1SE_ was based on the selection of 7 features, indicative of a more compact, yet comparably effective classifier. 6 of the 7 λ_1SE_ features were routine clinical measurements and the features retained in the regularised model are also listed in Table 2 for λ_1SE_.

### Model Evaluation on retained clinical validation data

**Figure 2:**
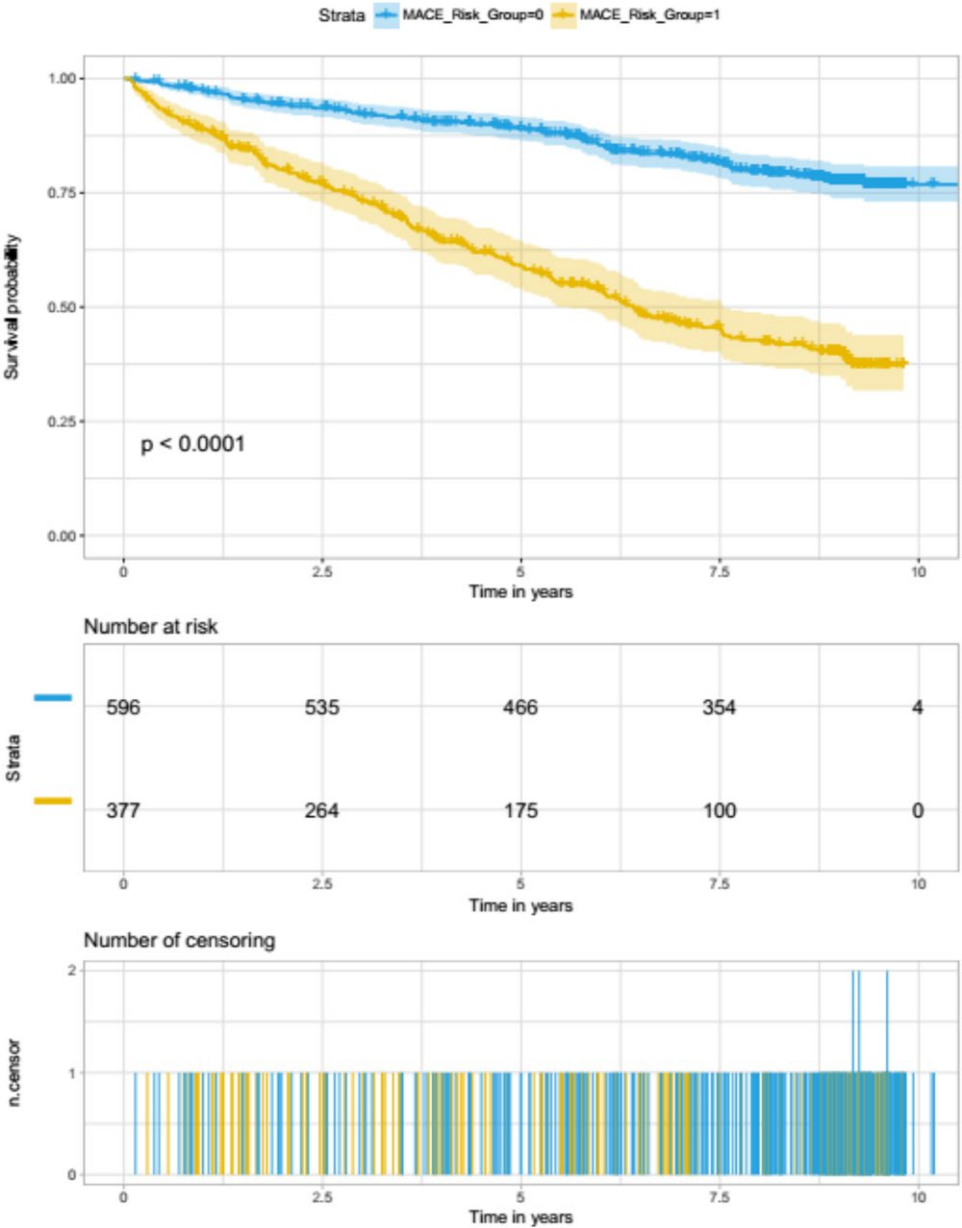
Kaplan-Meier curves for λ_min_-based model predictions and overall time-to-event analysis of the clinical validation set. Cases were stratified into two groups, high-risk and low-risk, using a pre-defined threshold (i.e., the mean predicted probability for the model-development set when λ_min_ was used in a 10 fold cross-validation).

**Figure 3:**
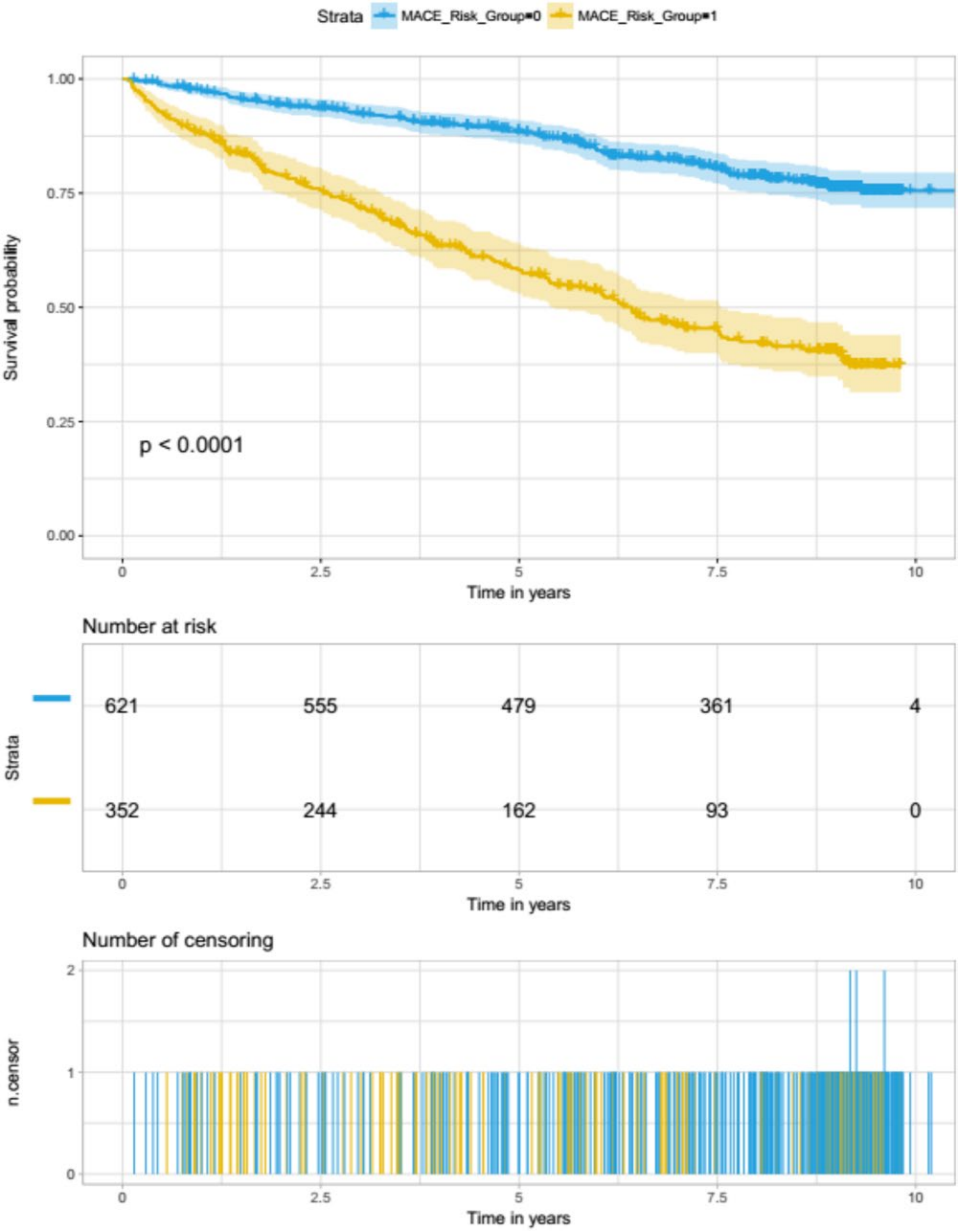
Kaplan-Meier curves for λ_1SE_-based model predictions and overall time-to-event analysis of the clinical validation set. Cases were stratified into two groups, high-risk and low-risk, using a pre-defined threshold (i.e., the mean predicted probability for the model-development set when λ_1SE_ was used in a 10 fold cross-validation).

The clinical validation set was evaluated using the regularised models based on the development set features. Figure 2 shows Kaplan-Meier curves for λ_min_-based model predictions and overall time-to-event (censoring point) of the clinical validation set. The time scale used was age; left-truncation was ensured by subtracting the age at imaging from age at event (or censoring). Cases were stratified into two groups, high-risk and low-risk, using a pre-defined threshold (0.47) in the model development stage. The numbers of participants in each risk category are listed beneath the curves. Figure 3 shows plots from the same procedure carried out using a λ_1SE_-based model.

The Kaplan-Meier curves generated using both models showed very similar patterns, whereby the number of individuals that belonged to the predicted high-risk group was consistently lower than half of those in the low-risk group, when observed at 2.5, 5, 7.5 and 10 years time points. In both cases, model predictions were highly associated with the MACE event (log rank p < 0.0001), indicating that the feature-sets used to train them captured strong signal for cardiovascular risk stratification.

Readers are referred to Supplementary material for a further analysis using only routine clinical measurements, age at imaging and sex.

### Bootstrap Analysis

In all 500 bootstrap trials, the model selected *age at imaging*, along with various groupings that always included features from each of the three main feature categories: retinal, genomic, and clinical (Table 3).

**Table 3:** Summary of bootstrap analyses.

Clinical features				Genomic features				Retinal features	
Feature	Frequency (%)	Feature	Frequency (%)	SNP	Frequency (%)	SNP	Frequency (%)	Subcategory	Frequency (%)
History of CVD	100	Duration of diabetes	54	rs3752728	95	rs687621	80	Tortuosity features	100
Diastolic blood pressure	99	Sex	44	rs12921187	89	rs2291435	78	Width gradient features	100
History of smoking	97	Cholesterol levels	31	rs4308	84	rs2014408	77		
High density lipoprotein	89	Systolic blood pressure	14	rs2048327	83	rs7136259	75	Branching point features	100
Glycated hemoglobin	82	Corrected systolic blood pressure	9	rs9549328	82	**Composite Score**	**Frequency (%)**	OD radius and/or OD-to-fovea	83
Number of blood pressure lowering drugs taken	71	Corrected diastolic blood pressure	3	rs34923683	81	CVD gene score	84	Fractal analysis features	82
				rs200999181	81	Alzheimer’s gene score	40	Zone B width features	71
Triglycerides	66					Blood pressure gene score	20		

All clinical features are listed with their corresponding frequencies. The three composite gene scores evaluated are included with their frequency distribution. Given the large number of SNPs included, only those selected with a frequency >75% have been included. Retinal features were evaluated as sub-categories given features within each sub-category were highly correlated. OD: optic disc; CVD: cardiovascular disease. Readers are referred to Supplementary Material for a detailed explanation of retinal features computed by VAMPIRE.

All clinical features are listed with their corresponding frequencies. Those selected at high frequencies across the trials (greater than 75%) were evidence of CVD before imaging, diastolic blood pressure, smoking history, high-density lipoprotein and glycated haemoglobin.

Genomic features included 343 SNPs and 3 composite gene scores. Of the 3 composite scores analysed, only the cardiovascular gene score was selected at a frequency greater than the defined threshold (>75%). Given the number of SNPs considered, only those exceeding the defined frequency threshold of 75% are highlighted (Table 4). Only 11 of the analysed SNPs exceeded the threshold. An interesting observation is that all 11 SNPs identified by bootstrap were also present in the list of 34 SNPs previously selected when building the model once using λ_min_. The 11 SNPs and their known phenotypic trait associations, as per the *Ensembl* genome browser, were rs12921187: diastolic blood pressure; rs4308: diastolic blood pressure; rs2048327: coronary heart disease; rs9549328: systolic blood pressure; rs34923683: pulse pressure; rs687621: cholesterol; rs2014408: depressive symptoms; rs7136259: coronary heart disease; as well as rs3752728, rs2291435, and rs200999181.

**Table 4:** Mean, median and standard deviation values of feature coefficients across the 500 regularised models. Individual features occurring at high frequencies (>75% threshold) are listed here.

Feature	Original scale	Mean β	Median β	Std. Dev. β
Age at imaging	years	0.03	0.03	0.01
History of smoking	[0, 1]	0.23	0.23	0.13
History of CVD	[0, 1]	1.05	1.05	0.14
Diastolic blood pressure	mmHg	−0.02	−0.02	0.01
High density lipoprotein	mmol/L	−0.23	−0.23	0.16
Glycated haemoglobin	mmol/mol	0.04	0.04	0.04
CVD gene score	raw scores	0.15	0.13	0.12
rs3752728	[0, 1, 2]	0.15	0.14	0.09
rs12921187	[0, 1, 2]	0.12	0.12	0.08
rs4308	[0, 1, 2]	−0.09	−0.08	0.07
rs2048327	[0, 1, 2]	0.08	0.07	0.07
rs9549328	[0, 1, 2]	0.10	0.09	0.08
rs34923683	[0, 1, 2]	0.27	0.24	0.23
rs200999181	[0, 1, 2]	−0.85	−0.90	0.65
rs687621	[0, 1, 2]	0.09	0.08	0.08
rs2291435	[0, 1, 2]	−0.07	−0.06	0.07
rs2014408	[0, 1, 2]	0.09	0.08	0.08
rs7136259	[0, 1, 2]	0.07	0.06	0.06

Note that the reported coefficients are relative to their corresponding features’ original scales e.g. one year increase in *age at imaging* corresponds to exp(0.03 +/− 0.01) increase in odds of developing a MACE outcome. Original scales are included in the table for reference. Gene variants are coded as 0, 1 or 2 representing the number of alternate alleles for the particular SNP the individual has inherited; coefficients are therefore the average per step going from 0 to 1 and 1 to 2. CVD: Cardiovascular disease. The CVD gene scores were included as raw values; coefficients are interpreted per unit step in the score.

Retinal features were grouped into the sub-categories described in Section 2.3.4. Features within each sub-category are highly correlated, and as such no individual retinal measurements were selected at high frequencies. We computed the frequencies with which the feature-set selected included at least one feature from each sub-category, and observed that all retinal sub-categories offered a highly robust signal: tortuousity sub-category (100%), width-gradient (100%), branching point (100%), OD-based (83%), fractal analysis (82%), and Zone B width (71%).

We finally computed the mean, median and standard deviation values of feature coefficients (β) across the 500 regularised models for individual features selected at highest frequencies (Table 4). This was carried out in an effort to illustrate the interpretability of our proposed approach. Whilst each bootstrap trial may offer a slightly different coefficient value, the coefficient sign is unlikely to change; utilising the coefficients of highly robust features can aid the answering of questions such as ‘*how does a one year increase in age affect the odds of an individual developing a MACE outcome?*’.

## Discussion

A cost-effective, non-invasive means of identifying high-risk individuals for MACE would be of tremendous value. In recent years, routine investigation of observable retinal characteristics has improved through advances in digital imaging, software capabilities, eye screening programmes, and wider availability through improved infrastructure at high street opticians. Previous evaluation of retinal features and cardiovascular risk has been limited. The cross-sectional, population-based Rotterdam study (n = 5,674) reported associations between wider venular diameter and atherosclerosis, inflammation and cholesterol^1^.

However, several studies have reported conflicting findings. The Cardiovascular Health Study reported associations between wide retinal venular calibre and high incidence of coronary heart disease (CHD) in both elderly women and men^2^, while other studies have found associations only in younger populations but not in elderly ones^23^. In light of these inconsistent findings, McGeechan and colleagues undertook a participant-level meta-analysis of over 22,000 participants from 6 studies^3^ and concluded that retinal vessel calibre changes (wider venules and narrower arterioles) associated with an increased risk of CHD in women but not men. However, that meta-analysis excluded studies on diabetic populations.

Other studies have also sought to improve the prediction of MACE, based on non-retinal data. McCarthy and colleagues^24^ developed linear models based on a 649 participants from the CASABLANCA study, incorporating a range of information on plaque erosion, acute phase reactants, inflammatory markers, and biomarkers of atherosclerosis. Model validation in an independent cohort illustrated the benefits and utility of integrating complementary clinical measurements from multiple sources to improve the prediction of individual MACE risk.

In this study, we investigated the combined potential of retinal parameters, genetic data and routinely collected clinical information for risk assessment of MACE in patients with type 2 diabetes. We used a regularisation approach in a supervised classification framework to develop a lasso-based predictive model. The model was developed and trained on a set of 2,918 participants and validated on an independent set of 973 participants. Lasso was similarly used to identify novel cancer biomarkers by Beck and colleagues^25^. The feature selection that underpins this approach is advantageous in that it summarises the multimodal features used into a single score (retinal vascular morphology, genetic data, clinical features). Additionally, the coefficients (β) associated with selected features can be used for interpreting the model and are relative to features’ original scales e.g. If the β coefficient associated with *age at scan* is 0.03, this means that a one year increase in age at scan corresponds to exp(0.03) increase in odds of developing a MACE outcome.

A suitable value for the lasso λ parameter can be determined through optimisation on the model-development set. Two values of λ were considered: **(a)** one that corresponds to the lowest binomial deviance (λ_min_), and **(b)** one that gives deviance within one standard error of (a) (λ_1SE_), achieving a similar performance whilst using a more compact set of features. A λ_min_ model selected 51 features whereas a λ_1SE_ model selected 7. The λ_min_-based model provides evidence for the utility of including retinal parameters, genetic data and clinical information to improve the accuracy associated with MACE risk stratification. However, comparable performance was achieved using mostly clinical information as identified by the λ_1SE_–based model (Fig. 1). These observations support previous findings from UK Biobank and EyePACS cohorts^4^.

It is important with any statistical feature selection method, lasso included, to obtain estimates of the relative robustness of selected and discarded features. Evaluation of features using *glmnet* does not imply that unselected features are weak; they may simply be highly correlated with those retained in the model. Furthermore, feature selection is sensitive to data sampling effects. This highlights the need for estimation of feature robustness, an essential step in biomarker discovery. Therefore, we performed a bootstrap analysis and identified the features or feature-sets occurring at high frequencies, using selection frequency as a proxy measure of robustness. Bootstrap analysis has been similarly used as a measure of robustness to investigate gene interaction, albeit using a different feature selection method^22^.

One feature that appeared in each bootstrap trial was age at imaging. Routine clinical features selected with high frequency for inclusion in the model (defined as >75%) included history of CVD, diastolic blood pressure, smoking history, HDL, glycated haemoglobin and genetic features (11 SNPs and cardiovascular gene score). Furthermore, bootstrap analysis revealed that some retinal parameters were always included when building the model, although individual features were not selected with the highest frequencies. Further analysis of the measures of tortuosity, vessel width and branching point sub-categories identified similar patterns, whereby different combinations of feature-sets were always selected (i.e. in every bootstrap there was always at least one tortuousity feature, at least one vessel-width feature, and at least one branching point feature). Retinal features from the Zone B vessel width, fractal analysis and OD-based sub-categories were selected in 71%, 82% and 83% of the trials, respectively.

In conclusion, this study yielded three main findings. Firstly, a multimodal classifier that was trained on retinal, genetic and routine clinical features was able to stratify risk of MACE in this cohort of patients with type 2 diabetes. This offers exciting future possibilities, such as rapid and inexpensive population screening technologies for the early detection of cardiovascular diseases. Secondly, we showed that a classifier trained mostly on routine clinical features was similarly able to stratify risk of MACE in this cohort. This suggests that risk of developing cardiovascular disease can manifest in various forms, and whilst retinal and genetic data can unveil such information on cardiovascular health, readily available clinical data can offer a complementary perspective. This is in line with state-of-the-art findings recently published on UK Biobank’s retinal and clinical data. Finally, we showed through a bootstrap analysis that all three sources of information (retinal, genetic, routine clinical) offer robust signal. In doing so, we also identified specific genetic variants that were selected at very high frequencies within each of the bootstrap models.

There are a number of limitations to our work. Firstly, we investigated only *semantic* retinal features, i.e. features capturing directly interpretable quantities of the vasculature. *Non-semantic* features ought to be included in the future, as candidates emerge from replicated, large deep-learning studies^4^, but ideally after their computation and clinical meaning have been clarified. Secondly, VAMPIRE retinal measurements are semi-automatic. While this reduces overall errors, it limits the number of images that can be measured in a given time period. The trade-off between accuracy and automation is currently under debate in the retinal image analysis community^26,27^. Thirdly, GoDARTS is a diabetic cohort. Hence, our findings complement those of similar studies on non-diabetic cohorts like UK Biobank and EyePACS but remain specific to the characteristics of our cohort. Replication on further diabetic cohorts is necessary. Fourthly, using one eye only per participant assumes sufficiently symmetric left-right measurements, an assumption *sub judice* in the recent literature^28^. Moreover, when carrying out missing feature imputation, the number of neighbours (k) used was set to 10. Investigating the optimal number of neighbours for use on this cohort using repeated cross-validation experiments, as well as investigating the optimal imputation strategy, would make interesting future work. Finally, longitudinal clinical information was represented by the median value of measurement across time; future work could use time series analysis to ensure more accurate data representation. In addition to the above, we plan to incorporate further retinal imaging modalities. Candidates being addressed in parallel studies including optical coherence tomography (OCT), OCT-angiography and ultra-wide-field-of-view imaging. The work will certainly require prospective analyses in clinical trials, but a reduction in mortality from CVD through early detection of risk by only a small percentage would represent several hundreds of thousands of lives saved annually worldwide, given that CVD represents 31%^29^ of all global deaths.

## Supplementary information

Supplementary Material
