# Supplementary material for: A multimodal approach to cardiovascular risk stratification in patients with type 2 diabetes incorporating retinal, genomic and clinical features
Source: Sci Rep. 2019 Mar 5;9:3591. doi: 10.1038/s41598-019-40403-1 (PMC6401035; doi:10.1038/s41598-019-40403-1)
Supplement: Supplementary file 1 — Supplementary Material [file 41598_2019_40403_MOESM1_ESM.docx]

**Supplementary Material**

**Article in *Scientific Reports***

A multimodal approach to cardiovascular risk stratification in patients with type 2 diabetes incorporating retinal, genomic and clinical features.

**Author names and affiliations**

Ahmed E. Fetit^a,^**^*^**

Alexander S. Doney^b^

Stephen Hogg^a^

Ruixuan Wang^a^

Tom MacGillivray^c^

Joanna M. Wardlaw^d^

Fergus N. Doubal^d^

Gareth J. McKay^e^

Stephen McKenna^a^

Emanuele Trucco^a^

^a^VAMPIRE project, Computer Vision and Image Processing Group, School of Science and Engineering (Computing), University of Dundee, Dundee, United Kingdom.

^b^Ninewells Hospital and Medical School, University of Dundee, Dundee, United Kingdom.

^c^VAMPIRE project, Centre for Clinical Brain Sciences, University of Edinburgh, Edinburgh, United Kingdom.

^d^Centre for Clinical Brain Sciences, University of Edinburgh, Edinburgh, United Kingdom.

^e^Centre for Public Health, Queen’s University Belfast, Belfast, Northern Ireland

**Correspondence to** afetit@dundee.ac.uk

**Appendix A – VAMPIRE 3.1 Retinal Feature Categories**

This appendix provides a description of the categories of retinal vascular feature measured by VAMPIRE 3.1 which were incorporated to our analysis.

**A.1 Reference System**

**A.1.1 Image zones**

The features are calculated according to a reference system that is based on the optic disc (OD) centre, the OD radius and the location of the fovea centre. The system divides the image in concentric regions centred on the OD centre (Supplementary Fig. S1):

1. OD: Area within 0.5 OD diameters from the OD centre.

2. Zone A: Annulus between 0.5 and 1.0 OD diameters from the OD centre.

3. Zone B: Between 1.0 and 1.5 OD diameters.

4. Zone C: Between 1.0 and 2.5 OD diameters.

5. Periphery: Area outside Zone C.

Regions relevant to vascular feature calculation with VAMPIRE are Zones B and C.


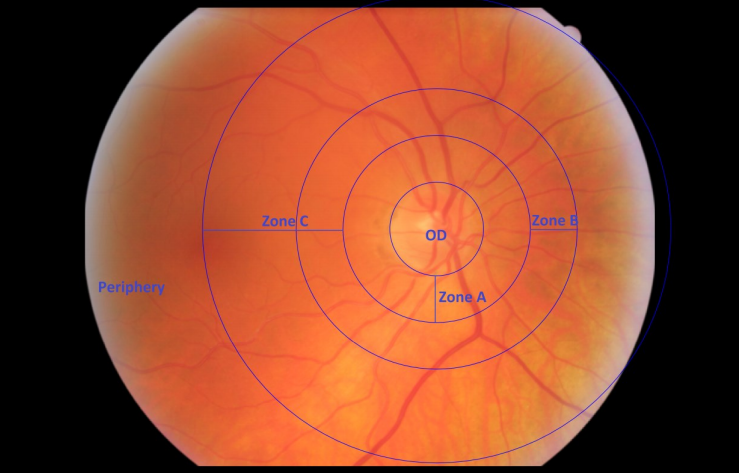
Supplementary Figure S1. Image zones according to a reference system based on the optic disc centre, the optic disc radius, and the location of the fovea centre.

**A. 1.2 Image quadrants**

The image is also divided into four quadrants that are numbered according to the following convention:

1. Supratemporal, 2. Inferotemporal, 3. Inferonasal, 4. Supranasal.

Quadrant numbering is consistent in left (L) and right (R) eyes in the sense that the quadrants are mirrored. For instance, Q1 is supratemporal for both L and R eyes. In the L image it will be the top-right quadrant, in the R image it will be the top-left.


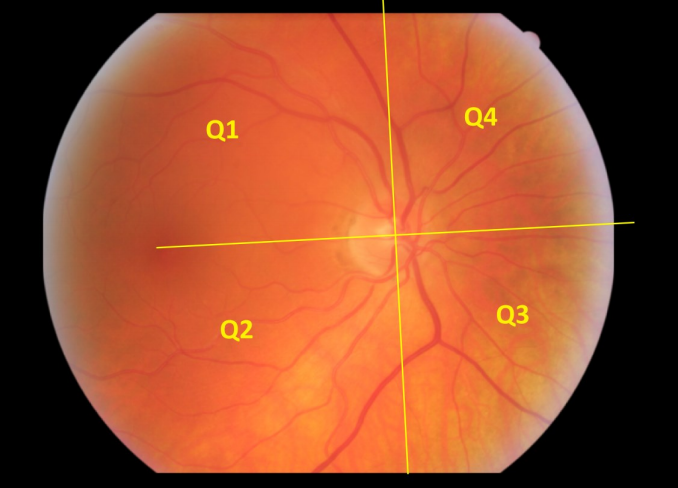


Supplementary Figure S2. Image quadrants.

**A.1.3 Vessel generation (tree structure)**

Vessel segments are grouped into tree structures; each tree is characterised by a **(i)** primary path, and **(ii)** a number of branches.

The tree’s primary path is referred to as the Generation-1 (G1) vessel. Branches attached to the G1 vessel are referred to as G2, and so on. Each tree is comprised of only one G1 vessel, but a variable number of G2, G3, G4, etc. branches.


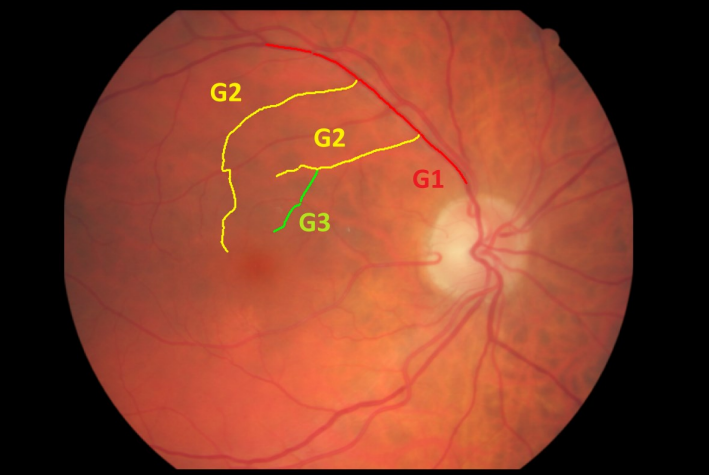


Supplementary Figure S3. Example of vessel generation labelling in a vessel tree.

**A.2 OD-based features**

These are:

- Odradius - Value of the OD radius in the original image (in pixels).
- Odfovea - Distance between the OD and the fovea centres in the original image (in pixels).

**A.3 Zone B width features**

Five width-related features are calculated within Zone B. In particular:

- CRAE - Central arterial equivalent (in pixels).
- CRVE - Central venular equivalent (in pixels).
- AVR - Arteriole to venule ratio (Patton et al., IOVS, 2006).
- BSTDa - Standard deviation of the widths of the arterioles used in the CRAE calculation (in pixels).
- BSTDv - Standard deviation of the widths of the venules used in the CRVE calculation (in pixels).

**A.4 Branching point features**

These features are calculated on correctly detected branching points located within Zone C; readers are referred to the work by Frost et al. (Transl. Psychiatry, 2013) for a good description of the formulae used to compute them.

The following features are width-related and are computed twice using different vessel-width estimation techniques; *Spline* (Cavinato et al., ISBI, 2013) and *Hermite* (Lupascu et al., MIA, 2013).

- BC a/v Hermite/Spline - Average values of branching coefficient for arterial / venular branching points identified in Zone C.
- AF a/v Hermite/Spline - Average values of asymmetry factor for arterial / venular branching points identified in Zone C.
- JE a/v Hermite/Spline - Average values of junctional exponent for arterial / venular branching points identified in zone C.
- LDR a/v Hermite/Spline - Average values of length to diameter ratio for arterial / venular branching points identified in zone C.

Additionally, the following are also computed:

- Num1stBa - number of first branching points in arterial vessel trees detected in Zone C.
- Num1stBv - number of first branching points in venular vessel trees detected in Zone C.

**A.5 Width gradient features**

These features are the gradients of the widths of the main arterial and venular vessel paths within Zone C, for each of the four image quadrants.

- Grad Q1-4 a/v Hermite - Gradient of width of the main arteriole / venule, in quadrants 1-4, estimated via Hermite.
- Grad Q1-4 a/v Spline - Same as above, but estimated via Spline.

**A.6 Fractal-based features**

These are obtained by carrying out fractal analysis on the binary centreline map of the arterial and venular vasculatures within Zone C. Readers are referred to the work by Stosic et al. (TMI, 2006) for a good description of the techniques.

- D0-2a – Fractal dimensions of the arterial vasculature.
- D0-2v – Fractal dimensions of the venular vasculature.

**A.7 Conventional tortuousity features**

Tortuosity features computed on the 6 largest arterioles and venules in Zone C, as per Annunziata et al. (MICCAI OMIA, 2014) .

- ZoneCATort Min/Max - Average, minimum and maximum values of tortuosity of the largest arterioles in Zone C.
- ZoneCVTort Min/Max - Average, minimum and maximum values of tortuosity of the largest venules in Zone C.

**A.8 Advanced tortuousity features**

These tortuosity features are computed for entire vessel paths that span from Zone B to Zone C. Values from different paths are combined by computing an average that is weighted according to corresponding path lengths (i.e. a shorter path would be assigned lower weight).

- tortQ1-4 a/v – Weighted average of the tortuosities of all vessels (arterioles or venules, respectively) paths in quadrants 1 to 4.
- tortQ1-4G1 a/v – Weighted average of the tortuosities of all G1 vessel paths in quadrants 1 to 4. Notice the difference with the previous set.
- tortImageG1 a/v - Weighted average of the tortuosities of all vessel paths in the entire image.
- tortQ1-4a Med/Std/Min/Max – Median, standard deviation, minimum and maximum values of the tortuosities of all arterial paths, respectively (regardless of G value) in quadrants 1 to 4.
- tortQ1-4v Med/Std/Min/Max – Median, standard deviation, minimum and maximum values of the tortuosities of all venular paths, respectively (regardless of G value) in quadrants 1 to 4.
- tortQ1-4G1a Med/Std/Min/Max – Median, standard deviation, minimum and maximum values of the tortuosities of all arterial paths of G1, respectively, in quadrants 1 to 4.
- tortQ1-4G1v Med/Std/Min/Max – Median, standard deviation, minimum and minimum values of the tortuosities of all venular paths of G1, respectively, in quadrants 1 to 4.
- tortImageG1 a/v Med/Std/Min/Max - Median, standard deviation, minimum and minimum values of the tortuosities of all arterial / venular paths of G1, respectively, in the entire image.
- tortMainPathQ1-4 a/v (real) – Tortuosity of the main arterial / venular path in quadrants 1 to 4.

**Appendix B- Separate Analysis using Clinical Measurements Only**

Following the results obtained with λ_1SE_-based model which showed that simply using readily available clinical information may provide good stratification, we carried out a separate analysis using only routine clinical measurements, age at scan and sex as features.

Testing this on the held-out clinical validation set indeed achieved accurate stratification (log-rank p value < 0.0001; Figure S4).

These observations were in line with the findings by Poplin et al. **[4]** who used datasets from UK Biobank and EyePACS cohorts, but did not include genomic information.

*
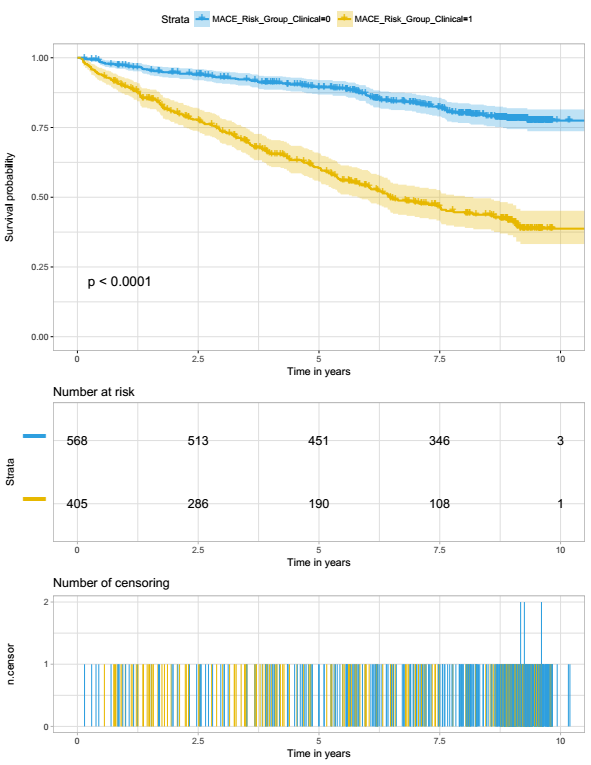
*

Supplementary Figure S4. Kaplan-Meier curves of the λ_min_-based model which was trained only on clinical information, age at scan and sex. Predictions and overall time-to-event on the clinical validation set are shown in the figure. Cases were stratified into two groups, high-risk and low-risk, using a pre-defined threshold; the mean predicted probability on the model-development set when λ_min_ was used in 10 fold cross-validation.
